# Increased Serum-Immunoglobulin G4 Levels in a 12-Year-Old Male Patient With Central Diabetes Insipidus

**DOI:** 10.7759/cureus.17362

**Published:** 2021-08-22

**Authors:** Takuro Kimura, Satoko Umino, Miyuki Kitamura, Shuichi Yatsuga

**Affiliations:** 1 Department of Pediatrics and Child Health, Kurume University School of Medicine, Kurume, JPN; 2 Department of Neonatology, St. Mary's Hospital, Kurume, JPN; 3 Department of Pediatrics, Aso Iizuka Hospital, Iizuka City, JPN

**Keywords:** increased serum igg4 levels, central diabetes insipidus, polydipsia, polyuria, igg4-related hypophysitis

## Abstract

Immunoglobulin G4 (IgG4)-related disorders are characterized by tissue hypertrophy due to IgG4-positive cell infiltration and increased serum IgG4 levels. IgG4-related hypophysitis (IgG4-RH) is characterized by pituitary hypertrophy, IgG4-positive cell infiltration, central diabetes insipidus, and increased serum IgG4 levels. IgG4-RH is diagnosed through diagnostic criteria. A few cases of IgG4-RH in children have been reported. We report a case of CDI with increased serum IgG4 levels that failed to meet the diagnostic criteria of IgG4-RH. The patient developed polyuria and polydipsia at age 11 and was diagnosed as having idiopathic CDI at age 12. The patient was not treated with steroids and is well controlled with antidiuretic hormones. It has been reported that pediatric IgG4-RH differs from that of adults in several respects. We believe that the pediatric IgG4-RH may not fit the diagnostic criteria of adults. There may be also other cases of increased serum IgG4 levels in pediatric patients with idiopathic CDI. We do not know if they are subtypes of IgG4-RH or if serum IgG4 levels are by chance raised in CDI, however, we report them here because IgG4-RH in children may be different from that in adults.

## Introduction

Immunoglobulin G4 (IgG4)-related disorders are characterized by enlargement and thickening of systemic organs (e.g., the pancreas, liver, gallbladder, lacrimal glands, salivary glands, and retroperitoneal space), high serum IgG4 levels, marked infiltration of IgG4-positive plasma cells, and fibrosis on histopathology [1, 2]. Hypophysitis can be classified as primary, when the cause is unknown and the inflammation is limited to the pituitary gland, and secondary when the cause is clear and is a symptom of systemic diseases [3], with IgG4-related hypophysitis (IgG4-RH) falling into this classification. IgG4-RH is diagnosed using established diagnostic criteria by Takagi et al. [3] and by Leporati et al. [4]

Although IgG4-RH may be infrequent, the symptoms show that central diabetes insipidus (CDI) and IgG4-RH generally appear in middle-aged and elderly males and young females [5], with limited reports of pediatric patients.

CDI is characterized by an insufficient secretion for AVP production from the hypothalamus-neurohypophyseal system after adequate stimulation [6]. There are various causes that can result in CDI, e.g., after pituitary surgery, trauma, and hypophysitis. The symptoms of CDI are a preference for cold drinks, drinking at night, presence of nocturia, polyuria, and polydipsia [6]. CDI due to hypophysitis is a rare condition, and CDI due to IgG4-RH is also rare. It has been reported that pediatric IgG4-RH may differ from that of adults in several respects. We believe that the pediatric IgG4-RH may not fit the diagnostic criteria for adults. There are also other cases of increased serum IgG4 in pediatric idiopathic CDI.

We do not know if they are subtypes of IgG4-RH or if IgG4 was accidentally raised in CDI, however, we report them here because IgG4-RH in children may be different from that in adults.

## Case presentation

IgG4-RH can be diagnosed using established diagnostic criteria formulated by Takagi et al. [3] (Table 1) and by Leporati et al. [4] (Table 2).

**Table 1:** Diagnostic criteria for IgG4-RH by Takagi et al.

I. Main symptoms
1. Symptoms due to mass lesion in pituitary gland, or those due to hypopituitarism.
2. Symptoms due to central diabetes insipidus.
II. Laboratory data and pathology
1. Decreased levels of one or more anterior pituitary hormones and those from the targeted organs.
2. Decreased responses of anterior pituitary hormones in stimulation tests.
3. Laboratory data that match the criteria of central diabetes insipidus.
4. Diffuse enlargement of pituitary gland and/or stalk on imaging.
5. Elevated levels of serum IgG4.
6. Infiltration with IgG4-positive plasma cells in pituitary biopsy samples.
7. Infiltration with IgG4-positive plasma cells in other involved organs.
III. Additional findings
1. IgG4-RH is more common in elderly men.
2. Pituitary mass and thickened stalk often respond to glucocorticoid therapy. Flares of Pituitary lesion during steroid tapering or development of new lesions in other organs should be monitored during a follow-up.
Definitive diagnosis of IgG4-RH is established when the following is fulfilled:
Any of the items in I and items 1, 2, 4 and 6 in II, or any of the items in I and items 3, 4 and 6 in II.
Probable diagnosis of IgG4-RH is established when the following is fulfilled:
Any of the items in I and items 1, 2, 4, and 7 in II, or any of items in I and items 3, 4, and 7 in II.
Possible diagnosis of IgG4-RH is established when the following is fulfilled:
Any of the items in I and items 1, 2, 4, and 5 in II, or any of items in I and items 3, 4, and 5 in II.

**Table 2:** Diagnostic criteria for IgG4-RH by Leporati et al.

Criterion 1: Pituitary histopathology. Mononuclear infiltration of the pituitary gland, rich in lymphocytes and plasma cells, with more than 10 IgG4-positive cells per high-power field.
Criterion 2: Pituitary MRI. Sellar mass and/or thickened pituitary stalk.
Criterion 3: Biopsy-proven involvement in other organs. Association with IgG4-positive lesions in other organs.
Criterion 4: Serology. Increased serum IgG4 (>140 mg/dL)
Criterion 5: Response to glucocortikoids. Shrinkage of the pituitary mass and symptom improvement with steroids.
Diagnosis of IgG4-RH is established when any of the following is fulfilled:
Criterion 1
Criterion 2 and 3
Criterion 2, 4, and 5

The patient was a 12-year-old Japanese male born at 37 weeks of gestation from a non-consanguineous marriage, with a birth weight of 2776 g and birth height of 47.3 cm. No abnormalities had been noted during his perinatal period. No atopic dermatitis, or immune/collagen diseases, as well as immune/collagen diseases in the family history, were noted. At 11 years of age, the patient developed polydipsia and polyuria, with increased water intake and nocturia consequently being noted, however, the patient had no fever, vomiting, constipation, or growth failure. Subsequently, the patient underwent a medical examination by a physician, who suspected diabetes insipidus. As such, the patient was referred to our department for medical treatment at 12 years old.

No concomitant symptoms, including headache or visual field disturbances associated with pituitary lesions, were observed. Blood tests revealed the following results: sodium, 143 mmol/L; plasma osmolality, 282 mOsm/kg; arginine vasopressin (AVP), 0.7 pg/mL; urine osmolality, 154 mOsm/kg; urine specific gravity, 1.004; and blood glucose, 91 mg/dL. The hypertonic saline infusion test showed elevated sodium (151 mmol/L) and plasma osmolality (299 mOsm/kg) with no change in AVP(0.9 pg/mL), urine osmolality (154 mOsm/kg), and urine specific gravity (1.004). Moreover, the pitressin tolerance test showed the following results: plasma osmolality, 299 mOsm/kg; urine osmolality, 591 mOsm/kg; and urine specific gravity, 1.014. Based on these results, a diagnosis of central diabetes insipidus (CDI) was established.

**Figure 1:**
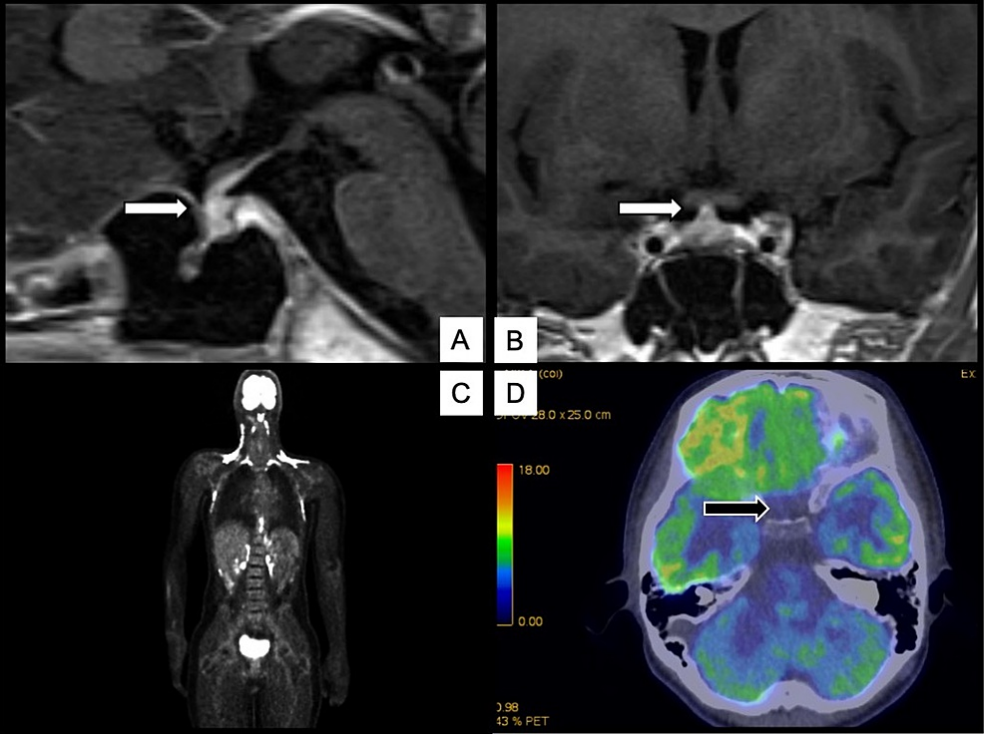
Enhanced T1-weighted MRI and FDG-PET The white arrow indicates the pituitary and stalk; no enlargement is observed (A, B). In the following two images, FDG-PET of the entire body (C) and brain (D) is shown. No accumulation of fluorodeoxyglucose (FDG) is observed. The black arrow indicates that there is no accumulation in the pituitary and stalk.

**Figure 2:**
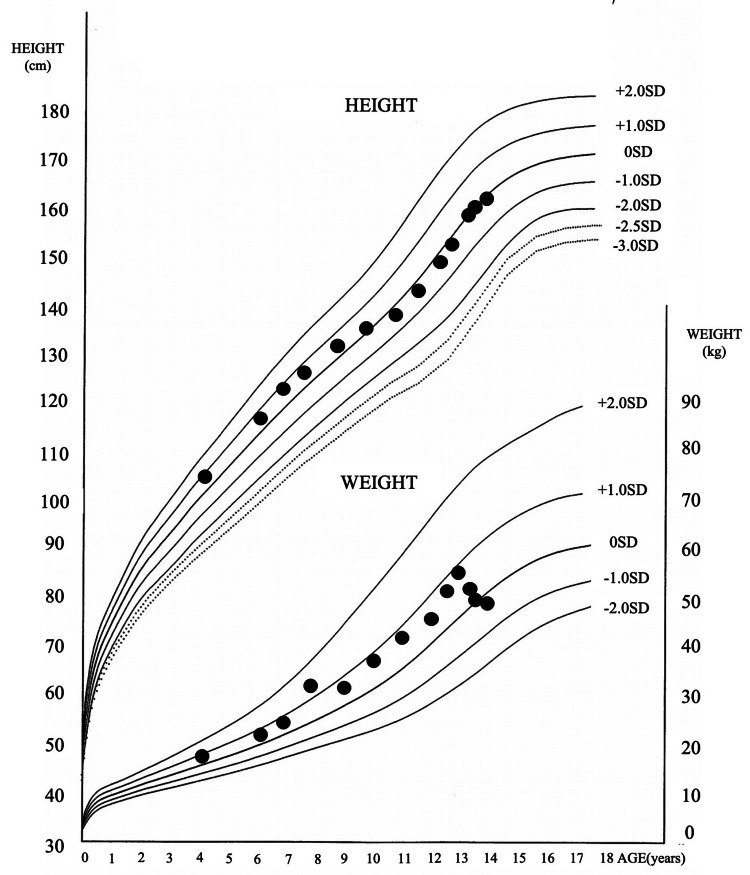
Cross-sectional growth charts. His height growth rate slightly decreased after age nine but was apparently normal after age 11. His body weight decreased after age 13.

Contrast-enhanced magnetic resonance imaging (MRI) of the head did not reveal a mass-like shadow extending from the pituitary stalk to the pituitary gland (Figures 1A-1B). The pituitary size was relatively swollen, albeit within normal range, with a height of 4.7 mm [reference range (rr) [7]; 4.22 ± 1.17 mm; mean ± SD], coronal width of 14.1 mm (rr; 13.04 ± 1.88 mm), a width of 5.88 mm (rr; 6.19 ± 1.21 mm), and a volume of 192.1 mm3 (rr; 172.0 ± 71.92 mm^3^). All anterior pituitary hormone function tests are summarized in Table 3. Although the baseline cortisol value was within the normal range, the peak cortisol value during the corticotropin-releasing hormone stimulation test was less than 18 μg/dL. The patient showed no symptoms of adrenal insufficiency. Anti-pituitary antibodies were negative, although anti-rabphilin-3A antibody was not tested. Moreover, alpha-fetoprotein (AFP) (1.3 ng/mL) and beta subunit of human chorionic gonadotropin-β (HCG) (<0.1 ng/mL) were within normal limits. Cerebrospinal fluid tests returned negative for placental alkaline phosphatase. The patient also had mildly elevated IgG4 levels (147 mg/dL), with the upper limit of normal for serum IgG4 levels and IgG concentrations being <135 and 1093 mg/dL, respectively. Antinuclear, anti-mitochondrial, and anti-smooth muscle antibodies, as well as MPO-ANCA and PR3-ANCA, were not assessed. A pituitary biopsy could not be performed due to the lack of consent of the patient’s family. Fluorodeoxyglucose-positron emission tomography (FDG-PET) showed no abnormal accumulation in the entire body, including the pituitary gland (Figures 1C-1D). As such, the patient was diagnosed with probable IgG4-RH. Oral desmopressin treatment was able to control the patient’s condition well, with no nocturnal enuresis having been observed. Given the absence of inflammation on FDG-PET, no symptoms associated with pituitary enlargement were present, while nocturnal enuresis had also improved. As such, glucocorticoids were not administered, and the patient was followed up. A growth curve is presented in Figure 2, which revealed a normal height gain but body weight loss after diagnosis and treatment. Thereafter, the patient’s course was evaluated through a head MRI every t months. Accordingly, anterior pituitary dysfunction and progressive enlargement of pituitary lesions had not been observed after two years, with pituitary size remaining within normal range: height (4.7 mm; rr [7], 5.24 ± 1.08 mm, mean ± SD), coronal width (13.7 mm; rr, 13.66 ± 1.79 mm), width (6.01 mm; rr; 6.59 ± 1.16 mm), and volume (193.4 mm; rr,237.17 ± 78.71 mm^3^).

**Table 3:** Anterior pituitary function test upon diagnosis Administration dose: arginine (0.5 g/kg), CRH (1.5 µg/kg), TRH (7 µg/kg), and GnRH (2.5 µg/kg).

	Arginine	CRH		TRH		GnRH	
	GH (mg/mL)	ACTH (pg/mL)	Cortisol (µg/dL)	TSH (µIU/mL)	PRL (ng/mL)	LH (mIU/mL)	FSH (mIU/mL)
Basal value	0.34	41.9	8.07	1.53	7.24	3.8	8.8
Peak value (min)	10.7 (60)	120 (30)	13.9 (60)	9.69 (30)	31.2 (30)	28.7 (30)	15.8 (60)

## Discussion

We herein report a case of a 12-year-old male patient with CDI and increased serum IgG4 levels. Symptoms indicating anterior pituitary dysfunctions (e.g., headache and/or visual field disturbance) or organ inflammation had not been observed. 

Although Leporati’s diagnostic criteria for IgG4-RH have been utilized worldwide [4], Japan has published their own diagnostic criteria for IgG4-RH in 2020 [3]. Considering that our case was a Japanese boy, we opted to utilize the Japanese diagnostic criteria [3]. In our case, I-2, II-3 and II-5 were fulfilled, suggesting no diagnosis with IgG4-RH. Pituitary biopsy could not be conducted given the parents’ refusal to collect samples of the pituitary lesion due to minimal enlargement of the pituitary gland, no anterior pituitary symptoms, and young age. The criteria for II-1 had not been satisfied by any anterior pituitary hormone levels, although the patient’s pituitary size was within normal limits. Pituitary biopsy remains the most definitive diagnostic test, despite being substantially invasive [8], while findings of IgG4-positive cells have not been histologically shown in 13.8% of patients [9]. Moreover, pediatric patients generally have smaller pituitary sizes than adults, making pediatric IgG4-RH may difficult to diagnose using either criteria.

One study showed that CDI was the most frequent endocrinological dysfunction in adults with IgG4-RH (70.2%) [9]. However, 17.9% of cases exhibited only CDI without anterior pituitary dysfunctions [10]. Another study showed that 50% of children with IgG4-RH had CDI alone [8]. Anterior and posterior dysfunctions have not been observed together in children and adolescents [11-13]. Although headache and visual field disturbance are uncommon symptoms among adults with IgG4-RH [10], headache has been observed in 50% of children [8,11,13]. Overall, 75% of adults showed other organ inflammation apart from the pituitary gland, whereas only 25% of pediatric cases showed the same [9]. Two young women previously reported [5] had no signs of clinical and radiological involvement of other organs. While IgG4-related inflammation may occur broadly in association with aging, pediatric cases may show milder severity compared to adult cases, with considerable differences in clinical features between the two. Similar to other diagnoses, such as lymphocytic infundibuloneurohypophysitis, pediatric cases of IgG4-RH may be underestimated. Notably, proactive measurement of serum IgG4 levels in conditions of hypophysitis or CDI of unknown causes may increase the number of detected pediatric IgG4-RH.

Generally, good response rates have been noted with glucocorticoid treatment for IgG4-RH (97.2% in adults) [10]. Alternatively, 2.8% of patients did not receive glucocorticoids given the presence of only CDI and a disease duration of >1 year from onset [10]. Our patient was not administered glucocorticoids due to the presence of only CDI, disease duration >1 year from onset, and no enhancement on FDG-PET over the entire body, including the pituitary and stalk, thus failing to fulfill the IgG4-RH criteria. We do not know how many pediatric patients exist with CDI with increased serum IgG4 levels that don't fulfill the diagnostic criteria for IgG4-RH.

Our patients have had no exacerbation of symptoms or complications despite not undergoing steroid therapy. In near future, it is necessary to study more cases of pediatrics CDI to determine the number of cases as well as the symptoms of increased serum IgG4 levels. 

The patient’s weight had decreased since the start of the AVP treatment for CDI. We consider the possibility that AVP treatment reduced stress by improving polydipsia and polyuria, especially at night, and ensuring good sleep quality.

## Conclusions

We report a case of pediatric CDI only with increased serum IgG4 levels that did not fulfill the diagnostic criteria for IgG4-RH. Although IgG4-RH in children is very rare, there may be cases of increased serum IgG4 levels in children with CDI only. Although the diagnostic criteria for IgG4-RH were not fulfilled in this case, careful follow-up to check for the appearance of IgG4-RD may be necessary in the future.
